# Mechanical Strength, Thermal Conductivity and Electrical Breakdown of Kenaf Core Fiber/Lignin/Polypropylene Biocomposite

**DOI:** 10.3390/polym12081833

**Published:** 2020-08-15

**Authors:** Harmaen Ahmad Saffian, Mohd Aizam Talib, Seng Hua Lee, Paridah Md Tahir, Ching Hao Lee, Hidayah Ariffin, Ainun Zuriyati Mohamed Asa’ari

**Affiliations:** 1 Institute of Tropical Forestry and Forest Products (INTROP), Universiti Putra Malaysia, UPM Serdang, Selangor 43400, Malaysia; Parida@upm.edu.my (P.M.T.); leechinghao@upm.edu.my (C.H.L.); ainunzuriyati@upm.edu.my (A.Z.M.A.); 2 Tenaga National Berhad Research (TNBR) Sdn Bhd., No.1, Lorong Ayer Itam, Kawasan Institut Penyelidikan, Kajang 43000, Selangor, Malaysia; aizam.talib@tnb.com.my

**Keywords:** kenaf core fibers, lignin, polypropylene, electrical breakdown, thermal conductivity

## Abstract

Mechanical strength, thermal conductivity and electrical breakdown of polypropylene/lignin/kenaf core fiber (PP/L/KCF) composite were studied. PP/L, PP/KCF and PP/L/KCF composites with different fiber and lignin loading was prepared using a compounding process. Pure PP was served as control. The results revealed that tensile and flexural properties of the PP/L/KCF was retained after addition of lignin and kenaf core fibers. Thermal stability of the PP composites improved compared to pure PP polymer. As for thermal conductivity, no significant difference was observed between PP composites and pure PP. However, PP/L/KCF composite has higher thermal diffusivity. All the PP composites produced are good insulating materials that are suitable for building. All PP composites passed withstand voltage test in air and oil state as stipulated in IEC 60641-3 except PP/L in oil state. SEM micrograph showed that better interaction and adhesion between polymer matrix, lignin and kenaf core fibers was observed and reflected on the better tensile strength recorded in PP/L/KCF composite. This study has successfully filled the gap of knowledge on using lignin and kenaf fibers as PP insulator composite materials. Therefore, it can be concluded that PP/Lignin/KCF has high potential as an insulating material.

## 1. Introduction

For the sake of environmental protection and economic viability, a renewed interest in using lignocellulosic materials or natural fibers as reinforcing agents for thermoplastic composites has been stimulated in the recent decades [1]. In the automotive industry, natural fibers were once popular as a reinforcing agent for thermosetting and thermoplastic composites in the 1920s. However, owing to their higher production cost and inferior performance, natural fibers has been gradually replaced by synthetic fibers [2]. Nevertheless, in recent years, rising awareness towards the environment well-being has encouraged people to adopt renewable resources, hoping for a diminution in greenhouse gases and carbon dioxide emissions. This is the time when people started to turn back to natural fibers [3]. Incorporation of natural fibers into the thermoplastic composite could bring a lot of advantages such as low density, improved Young’s modulus and renewability. However, drawbacks such as lower ultimate strength and higher water absorption are also the simultaneous consequences. Ahmad Saffian et al. [4] incorporated maleic anhydride modified lignin into kenaf core fiber-reinforced poly (butylene succinate) biocomposites and reported that the thermal stability of the composite was enhanced. However, lower tensile strength and modulus was observed.

Polypropylene (PP) is one of the most prevalently used thermoplastic polymers in the fabrication of natural fiber-reinforced polymer composite [5]. Despite the existence of a wide variety of thermoplastic polymers, PP stood out as a promising matrix owing to the fact that natural fiber started to degrade at temperatures around 210 °C. Therefore, only thermoplastic polymers that soften below this temperature are suitable for the production of natural fiber-reinforced polymer composites [6]. On the other hand, kenaf (*Hibiscus cannabinus* L.) is one of the popular natural reinforcing agents for polymer composites in Malaysia. It has a fast growing rate of 10 cm per day and is able to be cultivated four times a year, which has made kenaf an attractive natural fiber source [7]. Studies reported on PP reinforced with kenaf fibers has been reported extensively [8,9,10]. In fact, kenaf fiber has been proven to be more superior than flax, hemp, sisal and coir fiber as the polypropylene composites reinforced with kenaf exhibited higher specific modulus [11].

In natural fiber-reinforced polymer composites, dielectric properties of the produced composite are vital criteria that determine their final applications. To date, PP is still widely used in research and commerce due to its excellent characteristics and ease of fabrication process [10,12,13]. In high-voltage insulation systems, PP is one of the most commonly used polymeric material because of its good chemical resistance, mechanical properties and most importantly low dielectric loss, high dielectric resistivity and strength [14]. According to Hamzah et al. [15], the ability of a material to withstand the maximum voltage applied before the occurrence of breakdown failure is called dielectric breakdown strength. The dielectric withstands the voltage of the composite material to determine the quality and appropriateness of the chosen material as an insulation system. Generally, dielectric breakdown can be characterized as a sudden change in the resistance of the insulation material due to the applied voltage. Netnapa et al. [16] evaluated the effects of phosphorus-based non-halogenated flame retardant filler addition on the dielectrical breakdown strength of poly (L-lactic acid)-poly (lactic acid) microsphere/kenaf fiber composites. The authors reported an improvement in dielectrical breakdown strength when 2 wt % flame retardant filler was reinforced into the composite.

The ability of conducting heat (thermal conductivity) is a value-adding feature for polymeric composites. Thermally conductive polymeric composites possess lighter density, lower corrosion, oxidation, and chemical resistance as well as the flexibility to be tailor made to cater for various final applications, making it very suitable in replacing metal [17]. Heat sinks and packaging are two of the examples of the application of thermally conductive polymeric composites. However, studies on thermal conductivity of polymeric composites, particularly kenaf fiber-reinforced polymer composites, are rather limited from the literature. Liu et al. [18] produced rice straw fibers reinforced by polyurethane composites with excellent thermal insulating properties. The produced polymer composite showed promising potential to be used as insulating materials in buildings. Studies on thermal conductivity of kenaf fiber alone are also very scarce. The first study on the topic was reported by Gardner et al. [19] who evaluated the thermal conductivity of kenaf fiber before and after alkali treatment. In the same study, thermal conductivity and diffusivity of the unidirectionally oriented kenaf–epoxy composites reinforced with NaOH-treated and untreated kenaf fibers were assessed. Composite reinforced with NaOH-treated kenaf fibers possessed better thermal conductivity and diffusivity as interfacial contact between the fibers and epoxy matrix was enhanced after alkali treatment was applied to the kenaf fibers.

Lignin can be blended together with a wide variety of thermoplastic polymers in order to improve the performance and flowability of the resultant polymer composite [4]. Lignin is a green and renewable substance originate from plants. Commercially, lignin is a by-product generated from the pulping process and always exists in a huge quantity [20]. Regretfully, these by-products are not utilized effectively. Huang et al. [21] reported that addition of lignin could enhance the electrical breakdown voltage of PP and polyethylene (PE) composite. Nevertheless, reduction in mechanical strength was also observed. This phenomenon is caused by poor compatibility between nonpolar PP and PE and polar lignin. Therefore, addition of compatibilizer is necessary to improve the microphase structure of the blend system [21]. Polymeric methylene diphenyl diisocyanate (PMDI), an isocyanate compatibilizer, is often incorporated into the system [22]. Apart from that, lignin was also found to be able to increase the thermal conductivity of phenolic foam [23]. However, as mentioned in the above section, studies on the electrical properties and thermal conductivity of kenaf fiber-reinforced PP are very scarce. It opens up an opportunity for the research to fill the gap of knowledge. Kharade and Kale [24] reported that, with the assistance of a compatibilizer, the maximum content of lignin could achieve 30% and saw an improvement in the mechanical strength of the polyolefin materials treated with lignin. In addition, our previous study showed that incorporation of 30% lignin into kenaf core fiber-reinforced poly (butylene succinate) biocomposites has resulted in satisfactory tensile modulus and thermal stability [4]. Hence, in this study, 30% lignin was used for reinforcing PP/kenaf composites. Therefore, in this study, kenaf fiber-reinforced PP was fabricated and the effects of lignin incorporation on the mechanical strength, thermal conductivity and electrical breakdown of the resultant composite were evaluated.

## 2. Experimental

### 2.1. Materials

Polypropylene (TitanPro PM655) was acquired from Lotte Chemical Bhd, Johor, Malaysia. Kenaf core fiber (KCF) was purchased from National Kenaf and Tobacco Board (NKTB), Kelantan, Malaysia. KCF is supplied in powder form with size of 100 mesh. Lignin is low sulfonate content (Aldrich, St. Louis, MO, USA) purchased from Elta Globe Sdn. Bhd., Kuala Lumpur, Malaysia. Kraft lignin (L) used as filler had a particle size distribution in the range 10–200 µm. Its bulk density ranged from 400 to 500 kg m^3^ and its real density was around 1270 kg/m^3^. Polymeric methylene diphenyl diisocyanate (PMDI), as additives/compatibilizer was used to improve the performance and production of composites and was supplied by Elta Globe Sdn. Bhd., Kuala Lumpur, Malaysia.

### 2.2. Methods

**Figure 1:**
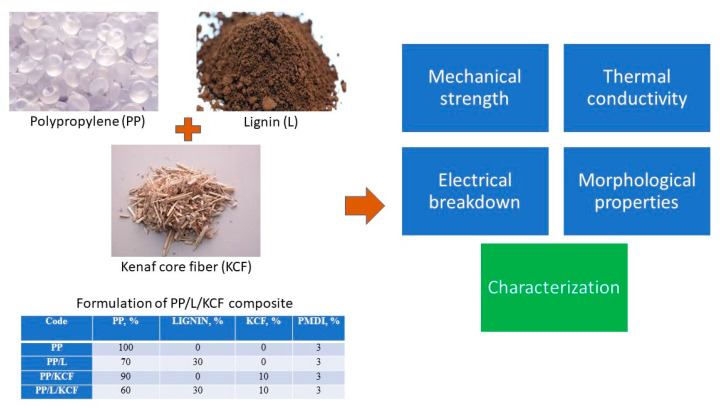
Schematic representation of the overall experiment.

PP/L/KCF with different fiber loading was prepared using compounding process. In compounding process, PP was melted in an internal mixer (Brabender, Duisburg, Germany). L and/or KCF was then added into the melt compound, followed by PMDI. The barrel parameters were set at 175 °C heating temperature and 120 rpm screw speed, respectively. The compounded materials were then crushed into smaller pieces for hot pressing process to form 300 × 300 × 2 mm^3^ plate. Hot pressing fabrication was conducted with a temperature of 175 °C. The crushed granules were pre-heated for 9 min and then quick pressed 5 times before being full pressed for 3 min. Then the heated materials were transferred to cold plate and press for 4 min. The formulation for PP composites fabrication are shown in Table 1. A schematic representation of the overall experiment is presented in Figure 1.

### 2.3. Characterization

#### 2.3.1. Physical and Mechanical Properties

Physical properties such as density and water absorption were evaluated based on the procedures specified in ASTM D570. For mechanical properties testing, a 2-mm thick plate produced through hot-press machine was cut into specimen shape according to ASTM D638 (tensile test) and ASTM D790 (flexural test). A mold with specific dimensions of 150 × 150 × 2 mm (length × width × thickness) was used for the sample preparation. Tensile and flexural tests were performed using a Universal Testing Machine (UTM, Instron- 3366) at a crosshead speed of 5 mm/min. For every set of formulations, 5 specimens were tested to determine the average properties. Prior to testing, the specimens were conditioned at room temperature in a desiccator for 24 h.

#### 2.3.2. Thermal Analysis

##### Thermogravimetric Analysis (TGA)

TGA was carried out by using TGA Q500 (Alzenau, Germany) from TA Instruments. Samples of 10 mg were used and heated in nitrogen environment (flow rate of 50 mL/min) with heating temperature started at 30 °C and ended at 800 °C and a temperature increment rate of 10°/min.

#### Differential Scanning Calorimetry (DSC)

In this study, DSC analysis were carried out by using a DSC Q20 (New Castle, DE, USA) from TA Instruments. 5 mg samples were placed in an aluminum crucible with a pin hole and heated in nitrogen environment (flow rate of 50 mL/min) with heating temperature started at 30 °C and ended at 400 °C with a temperature increment rate of 10°/min.

##### 2.3.3. Thermal Conductivity

The thermal conductivity of PP/L/KCF composites was determined using a Thermal Conductivity Analyser (λ-Meter EP500e, Dresden, Germany). The thermal conductivity measurement consists in applying variable heat flux in a block comprising a sample of size 150 mm × 150 mm × 3 mm thickness taken between two plates. The thermal conductivity is determined according to the standards ASTM C177.

#### 2.3.4. Electric Breakdown

The mechanism of electrical breakdown begins with the application of a strong electric field to the insulating material by a high voltage. Different levels of electric field are required for electrical breakdown voltage to occur. The test involves placing an extra-high voltage across the insulation sample until the breakdown of the material. The measurement was conducted with the test specimen immersed with oil and without oil (air) in accordance to IEC 60243-1. The samples initially were cut into a rectangular shape with dimension of 300 mm × 300 mm. Two electrodes consisting of two metal cylinders with diameter of 25 mm were placed between the insulation sample with one terminal connected to a high voltage source and the other one connected to a ground terminal. The measurement was conducted under a controlled environment in resonance test system by Haefely Trench RSK 100-1000-10 (Basel, Switzerland). The voltage applied started at 20 kV and increased gradually in 1-kV steps and was maintained for 20 s. The highest nominal voltage at which the insulation material withstood the force for 20 s without breakdown was considered as its electrical breakdown.

### 2.4. Statistical Analysis

One-way analysis of variance (ANOVA) was performed to evaluate the effect of formulations on the properties of PP/kenaf composites fabricated in this study. The mean values of each formulation were then separated by performing Tukey’s honest significant difference (HSD) test at *p* ≤ 0.05 level.

## 3. Results and Discussions

### 3.1. Physcial and Mechanical Properties

**Figure 2:**
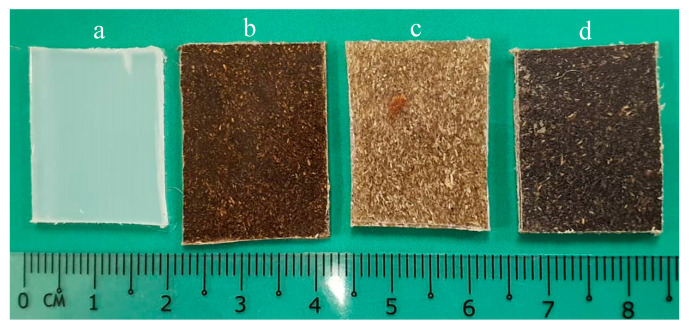
Apparent image of (**a**) PP, (**b**) PP/lignin (L), (**c**) PP/KCF, and (**d**) PP/L/KCF composites.

Figure 2 exhibits the apparent image of the PP and its composites produced in this study. Due to the native brown/black color of lignin itself, PP and its composites reinforced with lignin displayed a darker color [25].

The densities of the pure PP, PP/L, PP/KCF and PP/L/KCF were 0.91, 0.94, 0.93 and 0.98 g/cm^3^, respectively. On the other hand, the water absorption for these PP composites was 0.01, 0.05, 0.11 and 0.35%, respectively. Pure PP has the lowest water absorption. After the addition of lignin and kenaf fibers, the water absorption of the PP composites increased due to the hydrophilic nature of both the lignocellulosic materials [5]. The average value of tensile and flexural strength for PP/L/KCF composites fabricated in this study are shown in Table 2. Pure PP resin exhibited the highest tensile strength of 45.79 MPa. Reinforcement of 10 wt % kenaf fiber has slightly decreased the tensile strength of the PP composite to 44.17 MPa. The finding is contrary with the previous studies, who found incorporation of kenaf fibers had improved strength properties of the composites [26,27]. This scenario suggesting that poor interfacial bonding occurred between hydrophilic fillers and hydrophobic matrix, even though compatibilizers were applied [28]. On the other hand, the tensile strength of PP decreased significantly to 37.82 MPa after addition of 30 wt % lignin. This phenomenon is caused by the polar nature of lignin with has difficulty to compatible with nonpolar PP [21]. However, incorporation of both 10 wt % and 30 wt % lignin has resulted a PP composite with tensile strength comparable to that of pure PP. Tensile modulus of the PP/L/KCF composites mirrored that of tensile strength. With the assistance of a compatibilizer, PMDI in this case, the maximum lignin content of 30% could be added and bring improved mechanical properties to the composites [21]. As for flexural strength, pure PP has a flexural strength of 36.78 MPa. When 30 wt % and 10 wt % were added into the PP composites, the flexural strength of the PP composites increased significantly to 42.51 MPa and 43.16 MPa, respectively. The highest flexural strength of 43.55 MPa was observed in PP/L/lignin composite while the highest flexural modulus was recorded in pure PP.

### 3.2. Thermal Properties (TGA and DSC)

**Figure 3:**
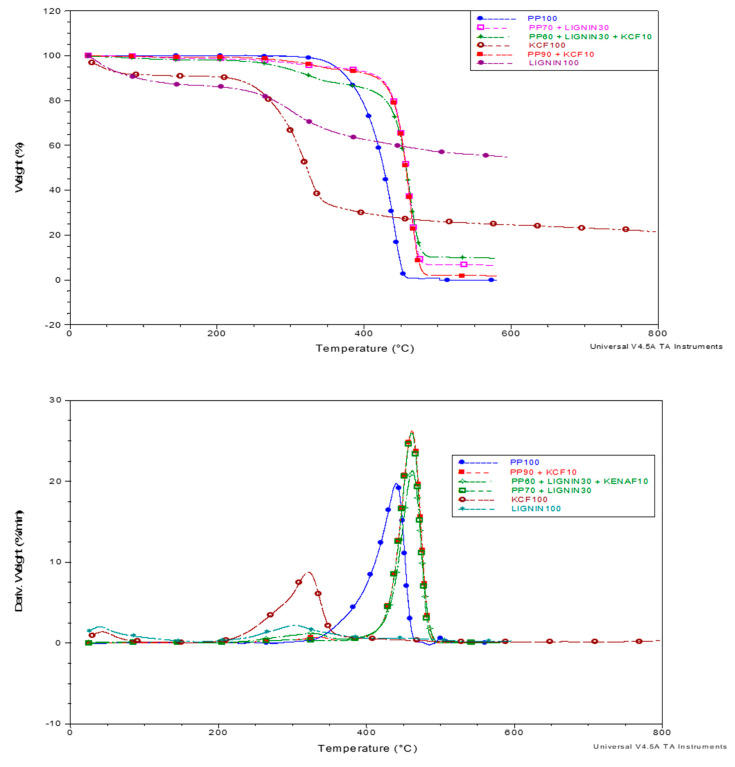
TGA (top) and DTG (bottom) curves of PP and its composites.

Table 3 shows the specific thermal decomposition temperature and residual mass after TGA testing while Figure 3 displayed the TGA and DTG curves across the temperature range. Slight mass reduction was observed at temperatures around 100 °C which could be attributed to the loss of moisture. Pure kenaf fiber (KCF100) and kenaf reinforced PP composites (PP/KCF and PP/L/KCF) exhibited higher mass loss at this stage, mainly due to the hydrophilic nature of kenaf fibers which tend to absorb higher amounts of moisture [29]. Pure PP has the highest onset decomposition temperature among all materials, ranging from 350.3 to 410.1 °C. The finding was in agreement with the previous study [30]. The polymer has higher onset temperature than lignocellulose materials, but extremely low mass residual at the end of testing [31]. On the other hand, lignocellulose materials show lower thermal stability and decomposed earlier at 139.3 and 255.2 °C for pure kenaf fiber and pure kraft lignin, respectively. Kenaf fiber consists of three main constituents which are cellulose, hemicellulose and lignin. Low thermal stability properties of hemicellulose constituents start to decompose, followed by cellulose and lignin, ranging from 150 to 380 °C according to a previous study [32]. Lignin constituents thermally decomposed at around 280–500 °C and were responsible for char formation (residual) [33]. These are similar to the results presented in this study; kenaf fiber decomposes at an earlier stage and has a lower residual compared to pure kraft lignin. The high thermal stability of lignin is because of its hindered structure, consisting of aromatic phenyl groups, which are very stable due to the overlapping of the p orbital.

The DTG curves shown in Figure 3 suggested that the maximum degradation of kenaf and lignin reinfored PP composites occurred at higher temperatures compared to that of pure PP. From the figure, one can see that pure kenaf fiber and kraft lignin has the the maximum degradation rate at around 300 °C while the maximum degradation rate for pure PP occured at around 450 °C. All the composites reinforced with either kenaf fiber or lignin or both have higher maxiumum degradation temperatures. PP/KCF, PP/L and PP/L/KCF have the identical maximum degradation temperature. However, PP/L/KCF has a lower degradation rate at the same temperature compared to PP/KCF and PP/L. Generally, incorporation of low thermal stability kenaf and/or lignin materials reduced onset temperature but reduced decomposition rate and resulted in higher mass residual at the same time.

**Figure 4:**
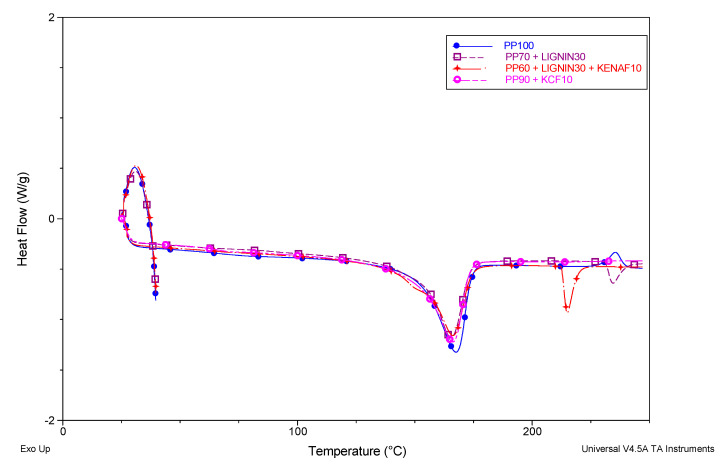
DSC curves of PP and its composites.

Table 4 shows the heat flows into the materials during heating by using DSC and Figure 4 illustrates the DSC curves of PP and its composites. A previous study has observed that the glass transition temperature (T_g_) of pure PP polymer at around 0 °C exhibited a brittle property [34]. It can be observed that the peak of T_g_ for PP composites is absent in Figure 4 as the T_g_ is below the temperature regime used in the DSC study. The insertion of kenaf or lignin component has resulted in the occurrence of two melting peaks where there is only one peak found in pure PP. Han et al. [35] attributed the existence of two melting peaks to the size distribution of crystalline lamella if the melting points are not produced due to the matrix polymorphism. The first melting point (T_m1_) occurred at 150 °C and 151.1 °C, respectively, for PP/KCF and PP/L. However, a drop of T_m1_ value for PP/L/KCF composite to 110.4 °C was observed. Besides, in comparison with the pure PP polymer and its composites, the DSC results show a slight decrease in second melting temperature (T_m2_) of PP/L/KCF composite specimen. This might be due to the fact that the kenaf fibers create hindrance in the melting of PP polymer [36]. On the other hand, incorporation of kenaf fiber and lignin together in PP polymer composite have possibility to reduce the total energy needed to break up the PP polymer chains in composite.

### 3.3. Thermal Conductivity

Table 5 listed the thermal conductivity, volumetric specific heat and thermal diffusivity of the PP composite fabricated in this study. Thermal conductivity of the PP composites ranged from 0.0854 to 0.0879 W/mK. Addition of lignin and kenaf fiber only slightly increased the thermal conductivity of the resultant composites. There is surprisingly very limited research reported on the thermal conductivity of kenaf reinforced PP composite. According to Asdrubali et al. [37], materials that possess thermal conductivity lower than 0.07 W/mK are categorized as thermal insulator. From the results obtained, on can see that the PP composites produced in this study are very close to that value. On the other hand, Karwa [38] stated that materials with thermal conductivity ranging from 0.03 to 3.0 W/mK are suitable for construction and building materials, where materials with thermal conductivity below 0.25 W/mK are often used for heat insulation. All the PP composites in this study fall into that range and therefore could be potentially used as a thermal insulator for buildings.

The ability of a material to store energy is called specific heat. Volumetric specific heat is therefore the heat needed by 1 m^3^ of material to alter its temperature by 1 K [37]. Pure PP and PP composite after being reinforced with 30 wt % lignin (PP/L) and 10 wt % kenaf fibers (PP/KCF) did not differ much with each other in term of volumetric specific heat. However, when both lignin and kenaf fibers were reinforced into the PP composites (PP/L/KCF), the volumetric specific heat decreased to 1.30 J/m^3^K compared to 1.57 J/m^3^K as recorded in pure PP. A similar trend was also observed for thermal diffusivity where there was no difference between PP, PP/L and PP/KCF composites. The thermal diffusivity of these three types composite was 0.055, 0.055 and 0.056 mm^2^/s, respectively. Interestingly, higher thermal diffusivity of 0.067 mm^2^/s was recorded in PP/L/KCF composites. The findings were in agreement with Asdrubali et al. [37] who stated that materials with high specific heat are often accompanied by low diffusivity values. PP/L/KCF composites have higher thermal diffusivity as they have lower specific heat value. PP/L/KCF composites have highest thermal diffusivity and thermal conductivity probably could be attributed to the crystalline materials in kenaf fiber that facilitate the thermal conduction and diffusion within the material [39]. Besides, composites with a higher density tend to have better thermal conductivity values as the thermal conductivity is highly dependent on the density [40]. As the density of PP/L/KCF composite was higher (0.98 g/cm^3^) compared to the other PP composites in this study, this may explain the reason the PP/L/KCF composite possess better thermal conductivity and diffusivity.

### 3.4. Electrical Breakdown

The electrical properties of PP/L/KCF composites board were determined by its electrical withstand strength under high voltage testing. The measurement was conducted with the test specimen immersed with oil and without oil. Starting with the applied voltage of 20 kV, the voltage was increased in incremental steps of 1 kV for 20 s until the breakdown occured. Table 6 displays the withstand voltage until flashover or breakdown of PP and its composite in air and oil. The results revealed that pure PP has higher withstand voltage of 35.70 kV and 42.10 kV tested in air and oil, respectively. After the addition of lignin and kenaf fibers, the values of withstand voltage of the PP composites decreased, both in air and oil. The withstand voltage of the tested sample in air ranged from 30.70 to 35.70 kV, where the pure PP showed the highest value followed by PP/L, PP/L/KCF and the lowest was recorded in PP/KCF. When tested in oil, the withstand voltage of PP/L/KCF and PP/KCF is almost comparable at 36.00 kV and 35.20 kV, respectively, whereas PP/L has the lowest withstand voltage of 24.12 kV. All the test samples have passed the withstand voltage requirement as stipulated in IEC 60641-3 with exception of PP/L composite tested in oil. The results suggested that the kenaf reinforced PP composite has high electrical strength properties and suitable to be used as dielectric or insulation material.

### 3.5. Morphology Properties of PP and Its Composite

**Figure 5:**
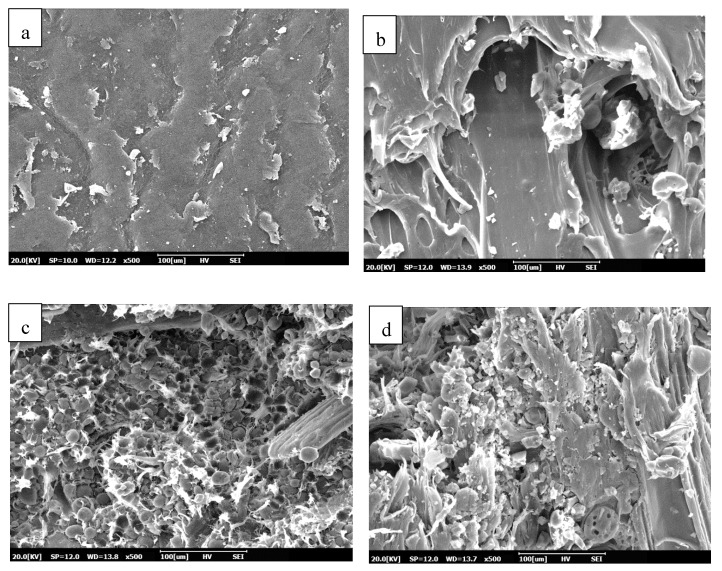
Morphology properties of (**a**) PP, (**b**) PP/L, (**c**) PP/KCF, and (**d**) PP/L/KCF composites.

Figure 5 shows the SEM micrographs of morphology structures of the pure PP, PP/L, PP/KCF and PP/L/KCF composites. Figure 5a exhibited the relatively smooth surface of pure PP. Figure 5b showed that rougher surface found on the PP composite blended with lignin. However, the surface was relatively smooth compared to PP/KCF and PP/L/KCF composites as shown in Figure 5c,d, respectively. As shown in Figure 5c, PP/KCF composite displayed rough interface and the existence of fiber pull-out. In Figure 5d, better compatibility between PP matrix, lignin and kenaf core fiber was observed as the resulting interface is rougher than that of PP/KCF composite. Han et al. [35] suggested that a smooth surface implied weak interactions and poor adhesion between the fibers and the polymer matrix. Therefore, it could be explained that the PP/L composite with a smoother surface has the lowest tensile strength as reported in the previous section. PP/KCF and PP/L/KCF exhibited significantly higher tensile strength than PP/L composite due to better interaction between fibers and matrix.

## 4. Conclusions

Lignin and kenaf core fibers have been reinforced into a PP polymer composite in this study. Generally, improved thermal stability and higher thermal diffusivity accompanied with comparable mechanical and electrical breakdown strength to that of pure PP was observed when both lignin and kenaf core fiber were incorporated into PP polymer. Tensile strength decreased when lignin and kenaf core fibers were incorporated into PP polymer. However, no significant different was recorded between pure PP and PP/L/KCF composites. Thermal stability of the PP composites was improved after incorporation of lignin and kenaf core fibers as indicated by increment in maximum degradation temperature. Investigation on thermal conductivity suggested that incorporation of kenaf core fibers and lignin did not yield significant changes in the conductivity of the PP composites. Nevertheless, PP/L/KCF exhibited lower volumetric specific heat and higher thermal diffusivity compared to the other PP composites. The results suggested that the PP composites fabricated in this study are suitable to be used as building insulating materials. On the other hand, all the PP composites surpassed the withstand voltage requirement as stipulated in IEC 60641-3, except for PP/L, suggesting that PP composites in this study are have potential to be used as dielectric or insulation material. In addition, SEM micrograph showed that better compatibility between PP matrix, lignin and kenaf core fiber was observed and therefore could be correlated well to the better mechanical strength of PP/L/KCF composite. This study has successfully filled the gap of knowledge on the thermal conductivity and electrical breakdown properties of kenaf core fiber- and lignin-reinforced PP composites. Despite that, future study is still needed in order to improve the interaction and adhesion between polymer matrix and kenaf core fibers and lignin.

## Figures and Tables

**Table 1:** Formulation of polypropylene (PP), Lignin and kenaf core fiber (KCF) compounding for board composites.

Code	PP, %	LIGNIN, %	KCF, %	PMDI, %
PP	100	0	0	3
PP/L	70	30	0	3
PP/KCF	90	0	10	3
PP/L/KCF	60	30	10	3

**Table 2:** Tensile and flexural properties of PP and its composites.

Composites	Tensile Strength (MPa)	Tensile Modulus (MPa)	Flexural Strength (MPa)	Flexural Modulus (MPa)
PP	45.79 ± 5.14 ^a^	1424.28 ± 142.10 ^a^	36.78 ± 4.20 ^b^	1975.43 ± 145.10 ^a^
PP/L	37.82 ± 4.30 ^b^	1241.28 ± 121.23 ^b^	42.51 ± 5.16 ^a^	1764.62 ± 132.15 ^ab^
PP/KCF	44.17 ± 5.10 ^a^	1409.35 ± 154.17 ^a^	43.16 ± 3.16 ^a^	1697.14 ± 132.16 ^b^
PP/L/KCF	44.60 ± 5.21 ^a^	1459.73 ± 156.10 ^a^	43.55 ± 4.12 ^a^	1801.66 ± 142.13 ^a^

Note: ^a,b^ Within the same properties row, mean values followed by different letters are significantly different at *p* ≤ 0.05.

**Table 3:** Thermogravimetric analysis (TGA) results of single component and PP composites.

Composites	Onset Temperature (T_on_), °C	Maximum Degradation Temperature (T_max_), °C	Residual, %
PP	350.3	410.1	1.1
Pure Kraft lignin	255.2	300.0	54.9
KCF	139.3	275.1	21.5
PP/L	283.5	441.5	1.9
PP/KCF	226.9	442.0	6.6
PP/L/KCF	275.8	472.3	9.7

**Table 4:** Differential scanning calorimetry (DSC) properties on PP and its composites.

Specimens	First Melting Temperature, T_m1_ (°C)	Second Melting Temperautre, T_m2_ (°C)
PP	-	164.5
PP/KCF	150.0	166.2
PP/L	151.1	166.2
PP/L/KCF	110.4	163.7

**Table 5:** Thermal conductivity and related properties of PP and its composites.

Composites	Thermal Conductivity (W/mK)	Volumetric Specific Heat (J/m^3^K)	Thermal Diffusivity (mm^2^/s)
PP	0.0854 ± 0.0011 ^a^	1.57 ± 0.04 ^b^	0.055 ± 0.003 ^b^
PP/L	0.0858 ± 0.0015 ^a^	1.54 ± 0.03 ^b^	0.056 ± 0.004 ^b^
PP/KCF	0.0877 ± 0.0017 ^a^	1.56 ± 0.05 ^b^	0.056 ± 0.003 ^b^
PP/L/KCF	0.0879 ± 0.0012 ^a^	1.30 ± 0.03 ^a^	0.067 ± 0.005 ^a^

Note: ^a,b^ Within the same properties row, mean values followed by different letters are significantly different at *p* ≤ 0.05.

**Table 6:** Withstand voltage (flashover) of PP and its composite material in air and oil.

Composites	Material in Air, kV	Current (A)	Material in Oil, kV	Current (A)
Standard IEC 60641-3	19 kV		30 kV	
PP	35.70	0.581	42.10	0.678
PP/L	35.03	0.573	24.12	0.396
PP/KCF	30.70	0.511	35.20	0.578
PP/L/KCF	30	0.586	36.00	0.590
